# Supplementary material for: MOF-Derived Ultrathin Cobalt Molybdenum Phosphide Nanosheets for Efficient Electrochemical Overall Water Splitting
Source: Nanomaterials (Basel). 2022 Mar 27;12(7):1098. doi: 10.3390/nano12071098 (PMC9000688; doi:10.3390/nano12071098)
Supplement: Supplementary file 1 [file nanomaterials-12-01098-s001.zip › nanomaterials-1652249-supplementary.pdf]

nanomaterials

MDPI

Supporting Information

MOF-Derived Ultrathin Cobalt Molybdenum Phosphide Nanosheets for Efficient Electrochemical Overall Water Splitting

Xiang Wang [1,2], Linlin Yang [1,2], Congcong Xing [1], Xu Han [3], Ruifeng Du [1,2], Ren He [1,2], Pablo Guardia [1], Jordi Arbiol [3,4] and Andreu Cabot [1,4,*]

[1] Catalonia Institute for Energy Research (IREC), Sant Adrià de Besòs, 08930 Barcelona, Spain; wxiang@irec.cat (X.W.); lyang@irec.cat (L.Y.); congcongxing@irec.cat (C.X.); ruifengdu@irec.cat (R.D.); renhe@irec.cat (R.H.); pguardia@irec.cat (P.G.)
[2] Departament d'Enginyeria Electrònica i Biomèdica, Universitat de Barcelona, 08028 Barcelona, Catalonia, Spain
[3] Catalan Institute of Nanoscience and Nanotechnology (ICN2), CSIC and BIST, Campus UAB, Bellaterra, 08193 Barcelona, Catalonia, Spain; xu.han@icn2.cat (X.H.); arbiol@icrea.cat (J.A.)
[4] ICREA, Pg. Lluis Companys, 08010 Barcelona, Catalonia, Spain
* Correspondence: acabot@irec.cat

Supplementary Materials:

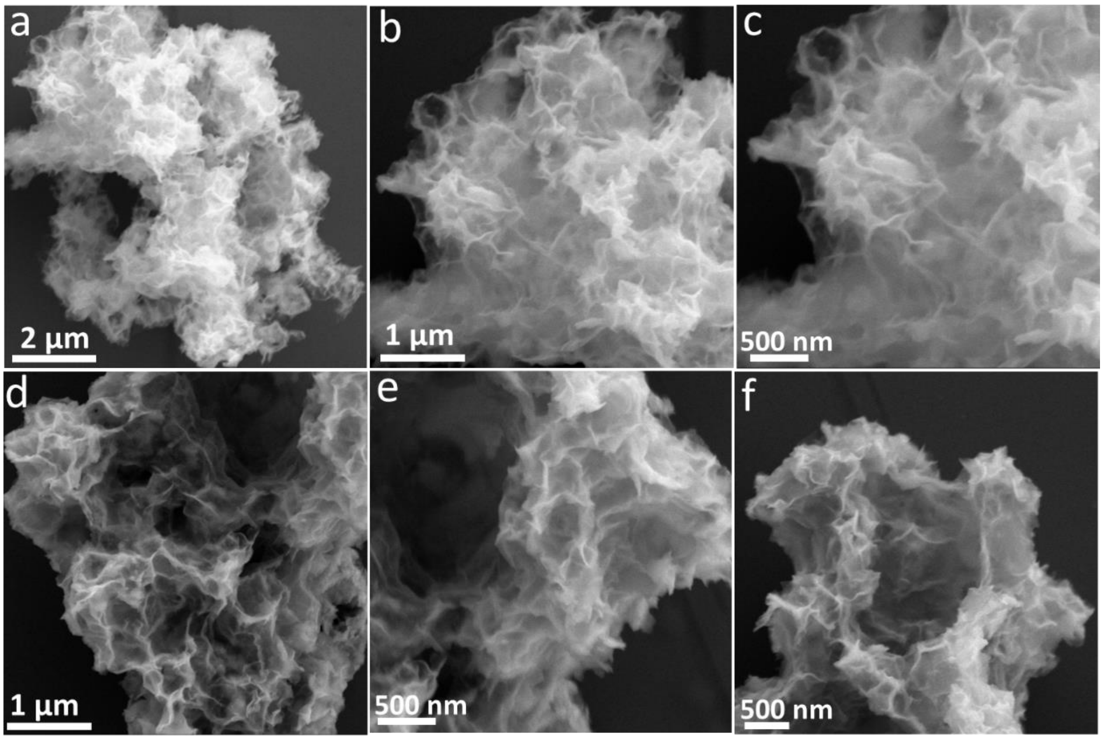

Figure S1. SEM images of CoMoP.

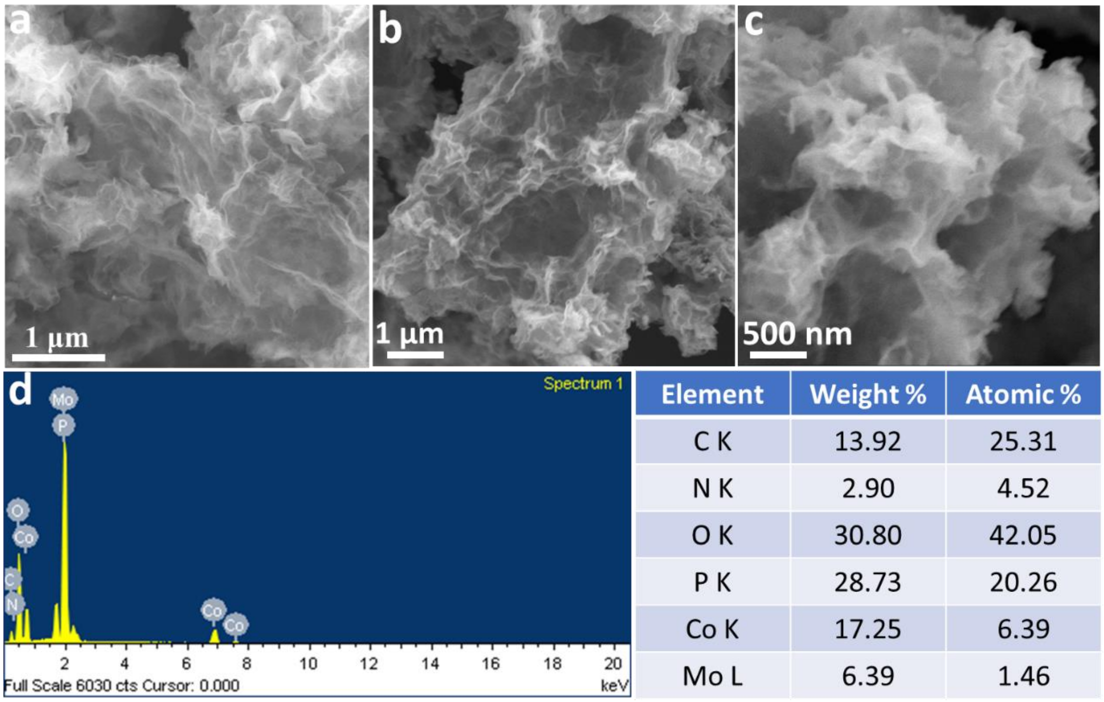

Element	Weight %	Atomic %
C K	13.92	25.31
N K	2.90	4.52
O K	30.80	42.05
P K	28.73	20.26
Co K	17.25	6.39
Mo L	6.39	1.46

Figure S2. (**a**) SEM image of Co–Mo MOFs. (**b**–**c**) SEM images and (**d**) EDX spectrum of CoMoP.

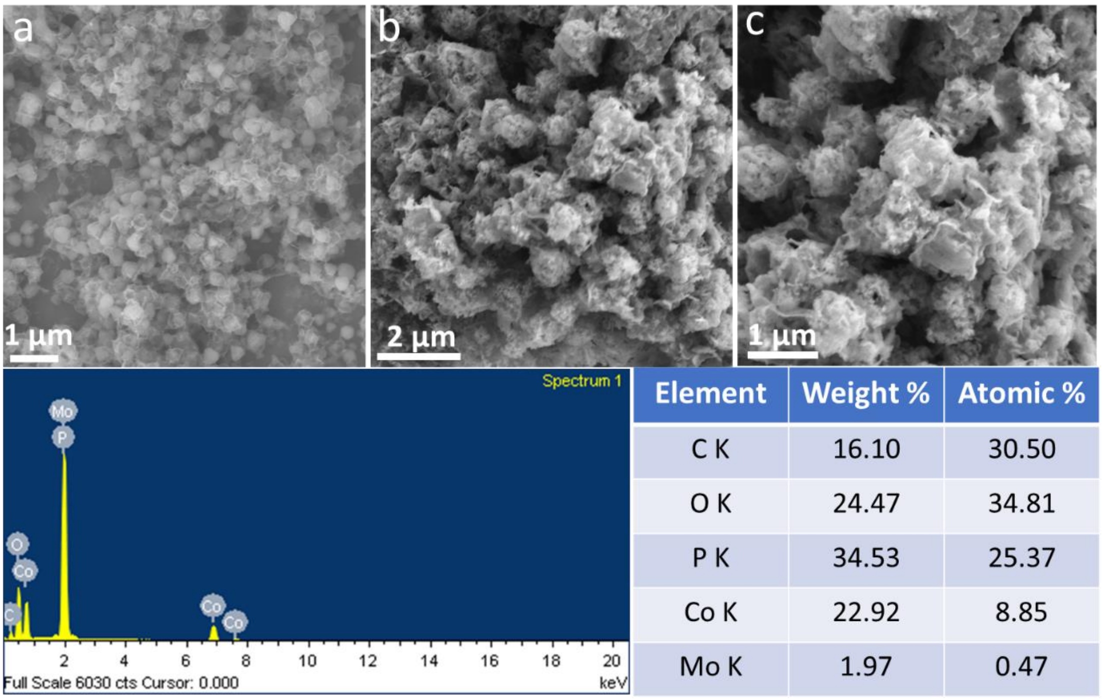

Element	Weight %	Atomic %
C K	16.10	30.50
O K	24.47	34.81
P K	34.53	25.37
Co K	22.92	8.85
Mo K	1.97	0.47

Figure S3. (**a**) SEM image of Na_2MoO_4-ZIF-67. (**b**–**c**) SEM images and (**d**) EDX spectrum Mo–CoP.

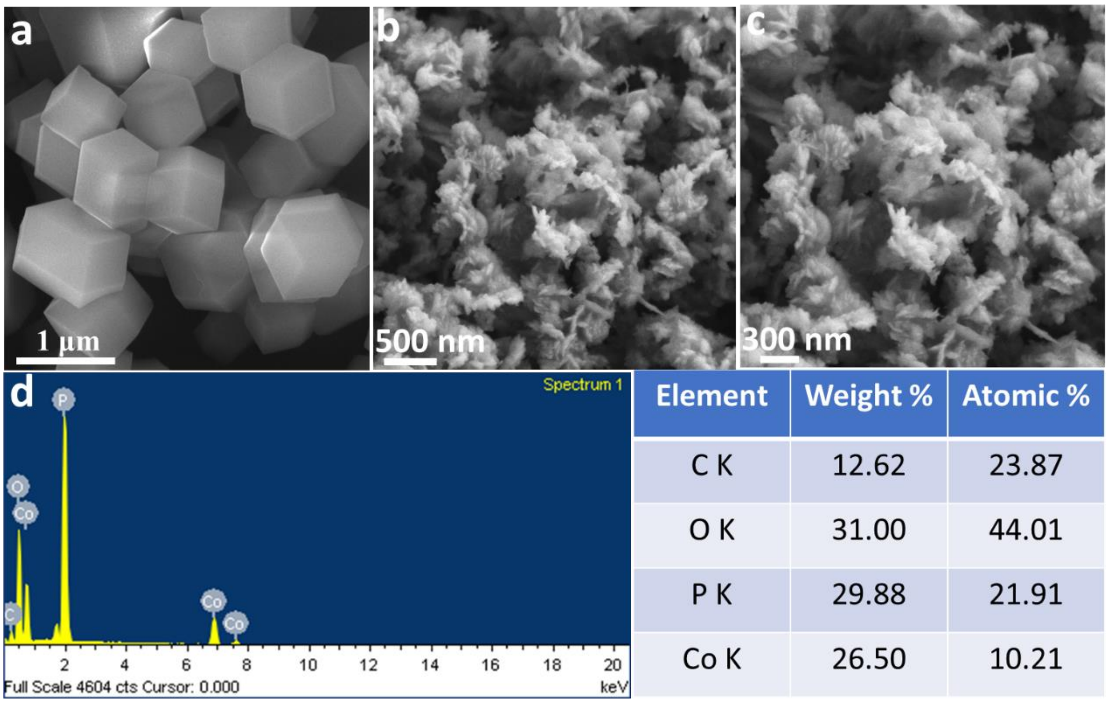

Element	Weight %	Atomic %
C K	12.62	23.87
O K	31.00	44.01
P K	29.88	21.91
Co K	26.50	10.21

Figure S4. (**a**) SEM image of ZIF-67. (**b–c**) SEM images and (**d**) EDX spectrum CoP.

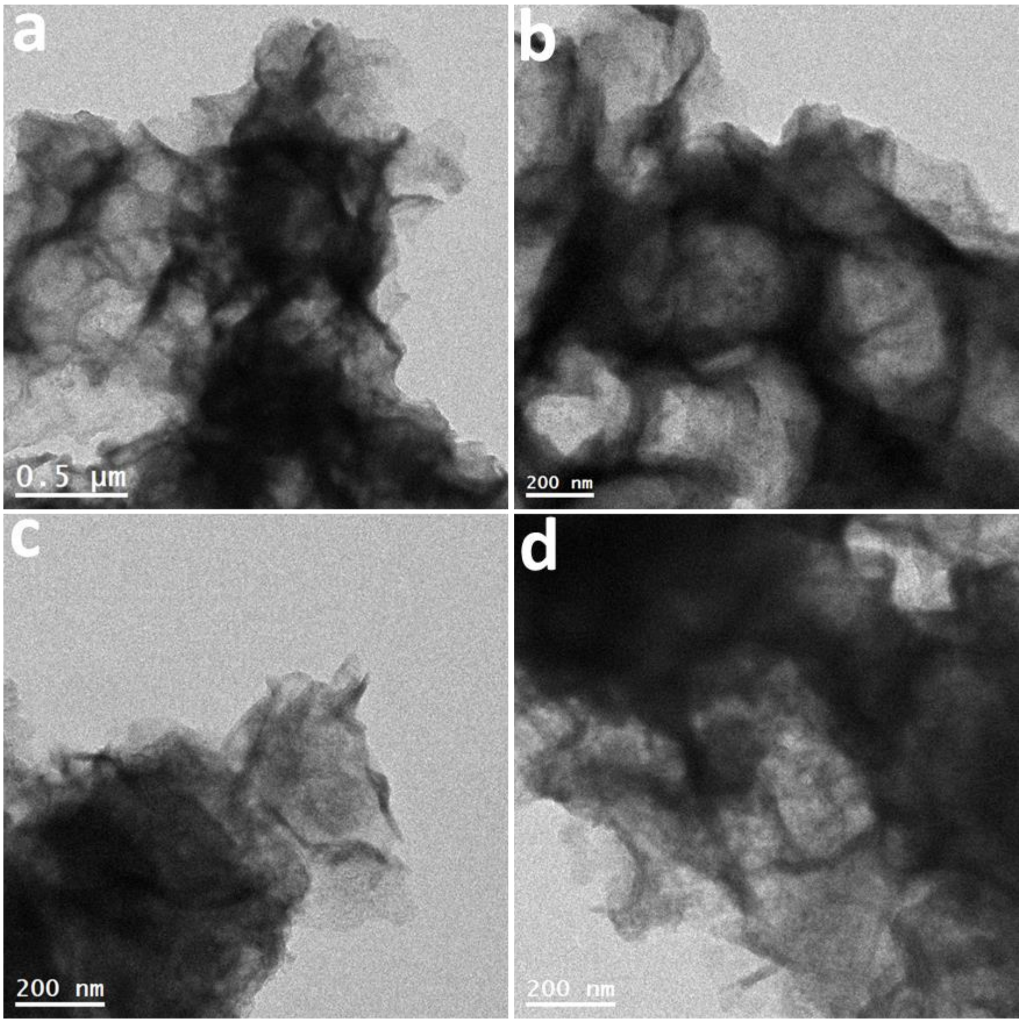

Figure S5. (**a–d**) TEM image of CoMoP.

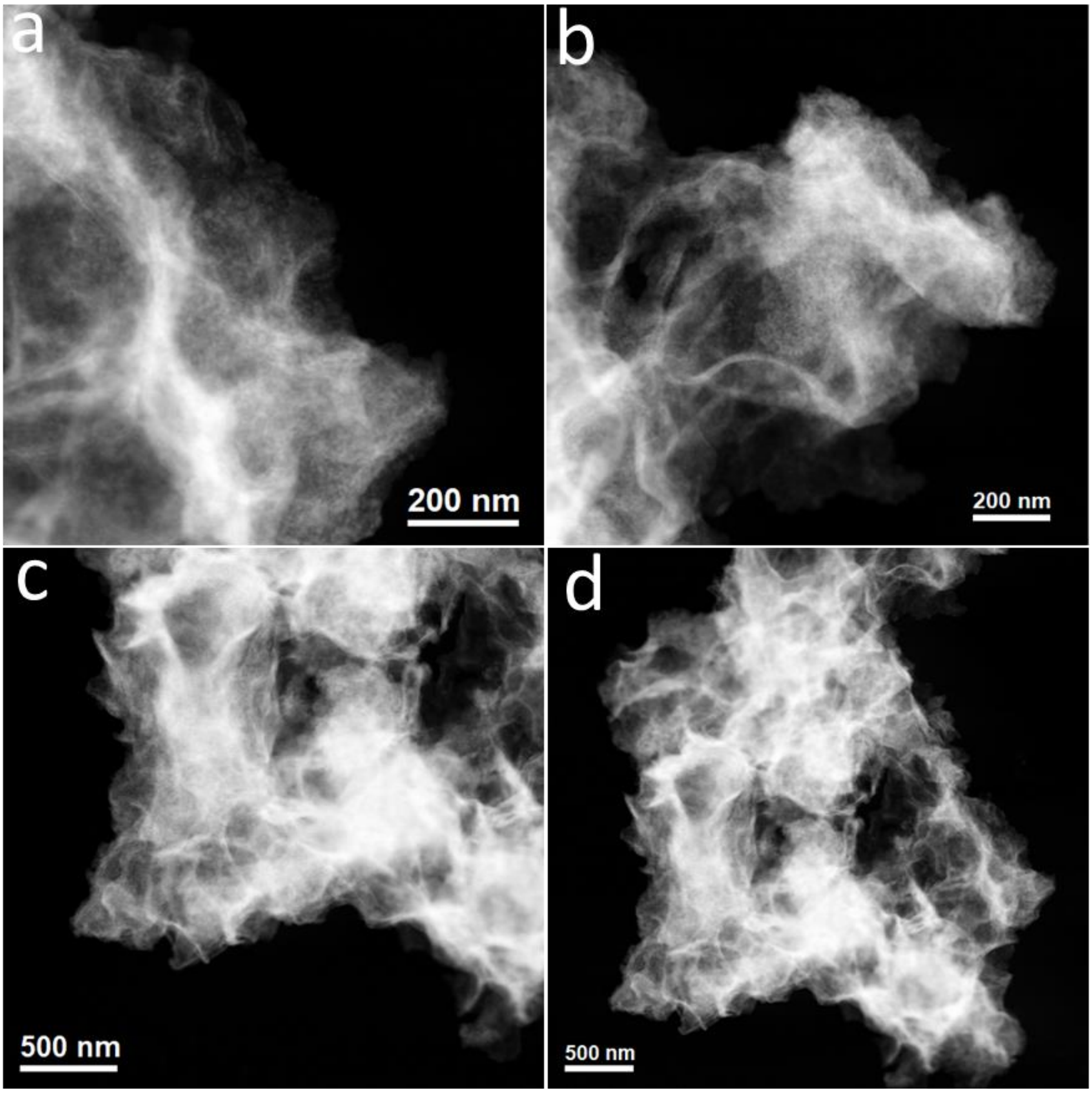

Figure S6. (**a–d**) HAADF-STEM micrographs of CoMoP.

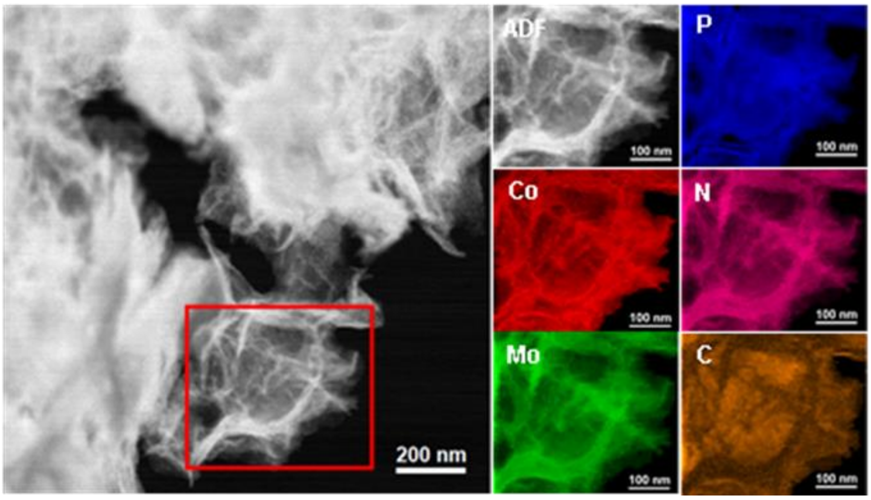

Figure S7. EELS chemical composition maps obtained from the red squared area of the STEM micrograph. Individual Co $L_{2,3}$-edges at 779 eV (red), Mo $M_{4,5}$-edges at 230 eV (green), P $L_{2,3}$-edges at 132 eV (blue), N K-edge at 401 eV (pink) and C K-edge at 284 eV (orange).

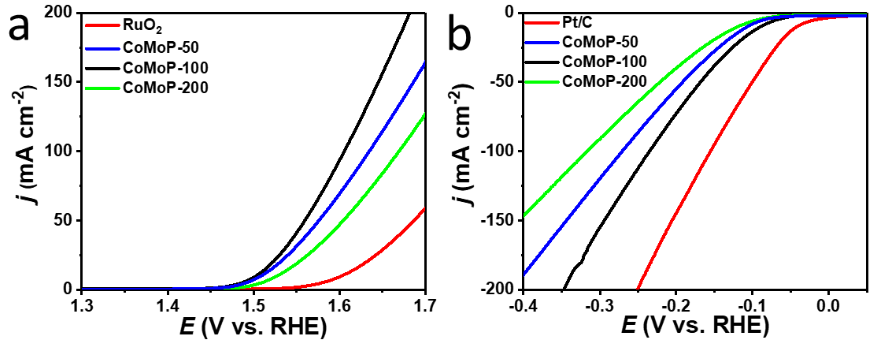

Figure S8. (**a**) OER and (**b**) HER polarization curves of CoMoP with different Mo content in 1.0 M KOH.

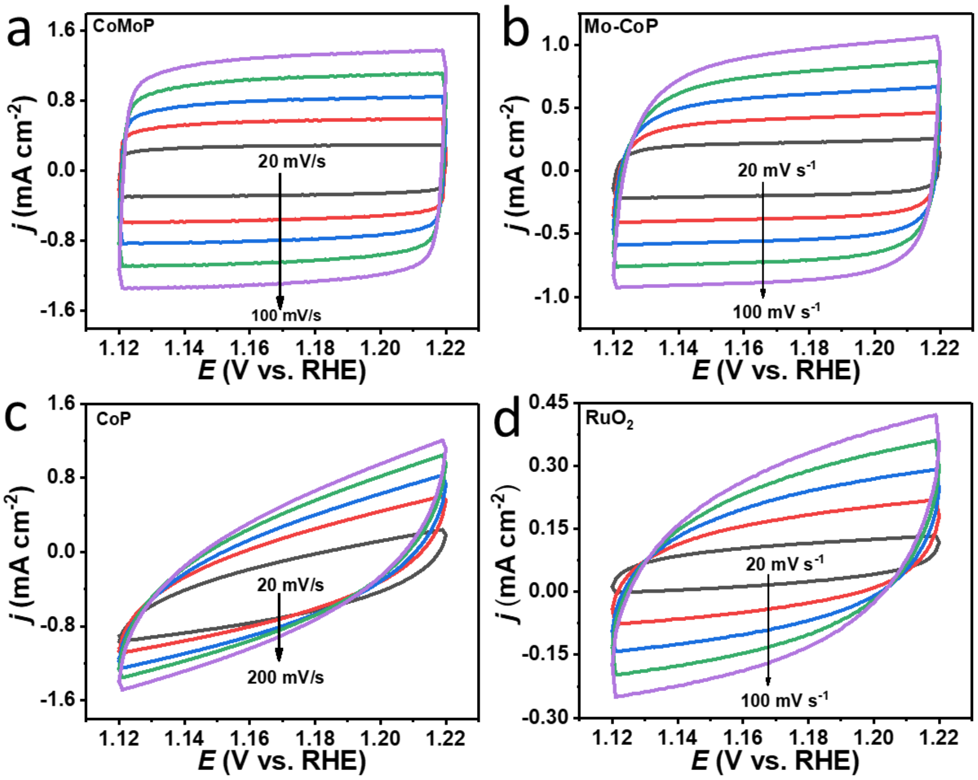

Figure S9. Cyclic voltammograms for (**a**) CoMoP; (**b**) Mo–CoP; (**c**) CoP and (**d**) RuO_2 in the non-faradaic region of 1.12–1.22 V vs. RHE at various scan rates.

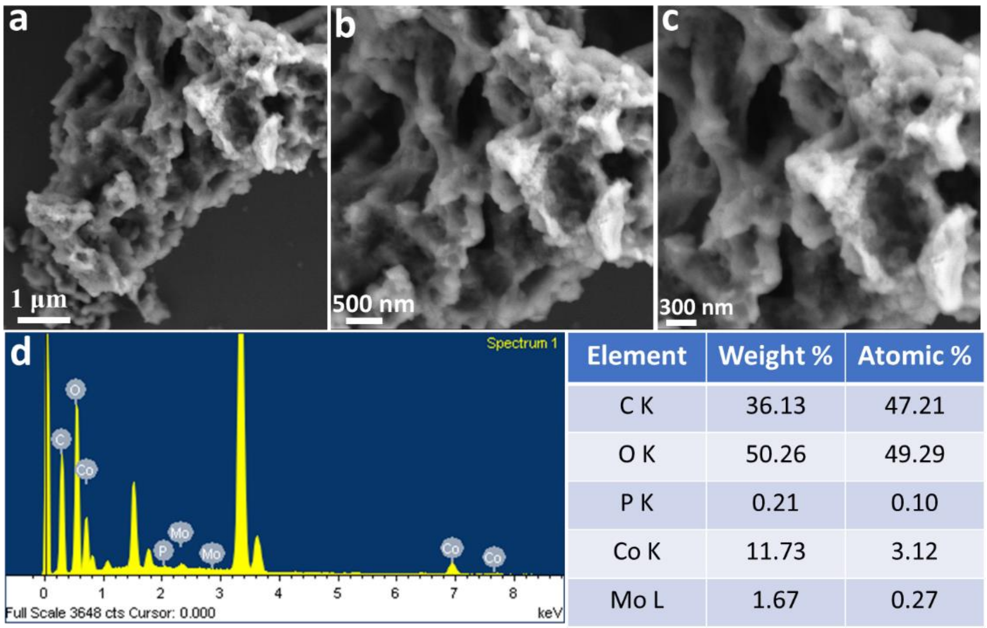

Element	Weight %	Atomic %
C K	36.13	47.21
O K	50.26	49.29
P K	0.21	0.10
Co K	11.73	3.12
Mo L	1.67	0.27

Figure S10. (**a**–**c**) SEM image and d) EDX spectrum of CoMoP after long term OER stability testing.

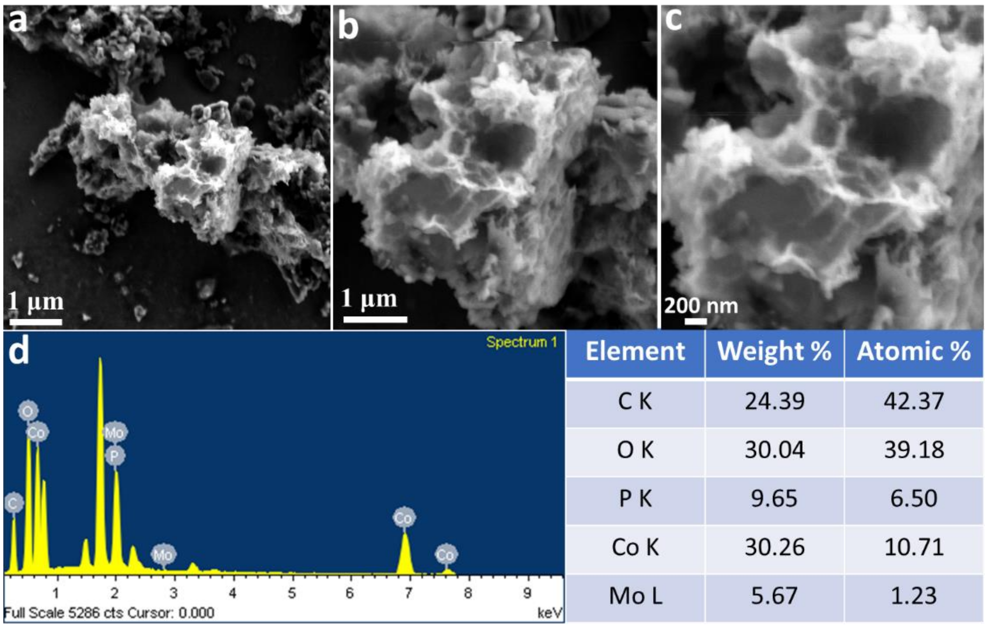

Element	Weight %	Atomic %
C K	24.39	42.37
O K	30.04	39.18
P K	9.65	6.50
Co K	30.26	10.71
Mo L	5.67	1.23

Figure S11. (**a**–**c**) SEM image and d) EDX spectrum of CoMoP after long term HER stability testing.

Table S1. Comparison of OER performance of CoMoP with some previously reported CoP-based catalysts in 1.0 M KOH solution.

Catalysts	Electrolyte	Overpotentials (mV)	Tafel Slope (mV/dec)	Reference
**CoMoP**	**1.0 M KOH**	**273**	**55**	***This work***
O-CoP nanosheets	1.0 M KOH	310	83	[1]
CoP/NCNHP	1.0 M KOH	310	70	[2]
Mo-CoOOH	1.0 M KOH	302	56	[3]
$CoP/CoCr_2O_4$	1.0 M KOH	290	52	[4]
CoP PNWs	1.0 M KOH	326	80	[5]
CoP/CNFs	1.0 M KOH	325	29	[6]

Table S2. Comparison of HER performance of CoMoP with some previously reported CoP-based catalysts in 1.0 M KOH solution.

Catalysts	Electrolyte	Overpotentials (mV)	Tafel Slope (mV/dec)	Reference
**CoMoP**	**1.0M KOH**	**89**	**70**	***This work***
NiFeP@N-CS	1.0 M KOH	186	112	[7]
NiCo-P	1.0 M KOH	169	68	[8]
$Ni_{0.85}Se$/RGO	1.0 M KOH	169	65	[9]
NCT@CoP@MoS_2	1.0 M KOH	195	74	[10]
Ce_1–CoP	1.0 M KOH	144	70	[11]
V-doped CoP	1.0 M KOH	235	91	[12]

Table S3. Comparison of OWS performance of CoMoP with some previously reported CoP-based catalysts in 1.0 M KOH solution.

Catalysts	Electrolyte	Potentials (V)	Reference
**CoMoP**	**1.0 M KOH**	**1.56**	***This work***
FeCoP UNSAs	1.0 M KOH	1.60	[13]
Cr-CoP/CP	1.0 M KOH	1.59	[14]
V-CoP@a-CeO_2	1.0 M KOH	1.56	[15]
NiCoP	1.0 M KOH	1.58	[16]
CoP–N/Co foam	1.0 M KOH	1.61	[17]
CoP@NPMG	1.0 M KOH	1.58	[18]
CoP/Co_2P	1.0 M KOH	1.57	[19]

References

1. Zhou, G.; Li, M.; Li, Y.L.; Dong, H.; Sun, D.; Liu, X.; Xu, L.; Tian, Z.; Tang, Y. Regulating the electronic structure of CoP nanosheets by O incorporation for high-efficiency electrochemical overall water splitting. *Adv. Funct. Mater.* **2019**, *30*, 1905252.
2. Pan, Y.; Sun, K.; Liu, S.; Cao, X.; Wu, K.; Cheong, W.-C.; Chen, Z.; Wang, Y.; Li, Y.; Liu, Y.; et al. Core–Shell ZIF-8@ZIF-67-Derived CoP Nanoparticle-Embedded N-Doped Carbon Nanotube Hollow Polyhedron for Efficient Overall Water Splitting. *J. Am. Chem. Soc.* **2018**, *140*, 2610–2618, https://doi.org/10.1021/jacs.7b12420.
3. Guan, C.; Xiao, W.; Wu, H.; Liu, X.; Zang, W.; Zhang, H.; Ding, J.; Feng, Y.P.; Pennycook, S.J.; Wang, J. Hollow Mo-doped CoP nanoarrays for efficient overall water splitting. *Nano Energy* **2018**, *48*, 73–80, https://doi.org/10.1016/j.nanoen.2018.03.034.
4. Saad, A.; Shen, H.; Cheng, Z.; Ju, Q.; Guo, H.; Munir, M.; Turak, A.; Wang, J.; Yang, M. Three-Dimensional Mesoporous Phosphide–Spinel Oxide Heterojunctions with Dual Function as Catalysts for Overall Water Splitting. *ACS Appl. Energy Mater.* **2020**, *3*, 1684–1693, https://doi.org/10.1021/acsaem.9b02155.
5. Zhang, M.; Ci, S.; Li, H.; Cai, P.; Xu, H.; Wen, Z. Highly defective porous CoP nanowire as electrocatalyst for full water splitting. *Int. J. Hydrogen Energy* **2017**, *42*, 29080–29090, https://doi.org/10.1016/j.ijhydene.2017.09.171.
6. Xie, X.-Q.; Liu, J.; Gu, C.; Li, J.; Zhao, Y.; Liu, C.-S. Hierarchical structured CoP nanosheets/carbon nanofibers bifunctional electrocatalyst for high-efficient overall water splitting. *J. Energy Chem.* **2021**, *64*, 503–510, https://doi.org/10.1016/j.jechem.2021.05.020.
7. J.C. Hei, G.C. Xu, Wei, L. Zhang, H. Ding, D.J. Liu. NiFeP nanosheets on N-doped carbon sponge as a hierarchically structured bifunctional electrocatalyst for efficient overall water splitting. *Appl. Surf. Sci.* **2021**, *549*, 149297.

8. C. Shuai, Z.L. Mo, X.H. Niu, P. Zhao, Q.B. Dong, Y. Chen, N.J. Liu, R.B. Guo Nickel/cobalt bimetallic phosphides derived metal-organic frameworks as bifunctional electrocatalyst for oxygen and hydrogen evolution reaction. *J. Alloys Compd.* **2020**, *847*, 156514.
9. Liu, G.; Shuai, C.; Mo, Z.; Guo, R.; Liu, N.; Niu, X.; Dong, Q.; Wang, J.; Gao, Q.; Chen, Y.; et al. The one-pot synthesis of porous $Ni_{0.85}Se$ nanospheres on graphene as an efficient and durable electrocatalyst for overall water splitting. *New J. Chem.* **2020**, *44*, 17313–17322, https://doi.org/10.1039/d0nj04197a.
10. Zhang, C.-L.; Xie, Y.; Liu, J.-T.; Cao, F.-H.; Cong, H.-P.; Li, H. 1D Core−Shell MOFs derived CoP Nanoparticles-Embedded N-doped porous carbon nanotubes anchored with MoS2 nanosheets as efficient bifunctional electrocatalysts. *Chem. Eng. J.* **2021**, *419*, 129977, https://doi.org/10.1016/j.cej.2021.129977.
11. Li, J.; Zou, S.; Liu, X.; Lu, Y.; Dong, D. Electronic Modulation of CoP by Ce Doping as Highly Efficient Electrocatalysts for Water Splitting. *ACS Sustain. Chem. Eng.* **2020**, *8*, https://doi.org/10.1021/acssuschemeng.0c01193.
12. Qin, J.-F.; Lin, J.-H.; Chen, T.-S.; Liu, D.-P.; Xie, J.-Y.; Guo, B.-Y.; Wang, L.; Chai, Y.-M.; Dong, B. Facile synthesis of V-doped CoP nanoparticles as bifunctional electrocatalyst for efficient water splitting. *J. Energy Chem.* **2019**, *39*, 182–187, https://doi.org/10.1016/j.jechem.2019.01.022.
13. Zhou, L.; Shao, M.; Li, J.; Jiang, S.; Wei, M.; Duan, X. Two-dimensional ultrathin arrays of CoP: Electronic modulation toward high performance overall water splitting. *Nano Energy* **2017**, *41*, 583–590, https://doi.org/10.1016/j.nanoen.2017.10.009.
14. Li, W.; Jiang, Y.; Li, Y.; Gao, Q.; Shen, W.; Jiang, Y.; He, R.; Li, M. Electronic modulation of CoP nanoarrays by Cr-doping for efficient overall water splitting. *Chem. Eng. J.* **2021**, *425*, 130651, https://doi.org/10.1016/j.cej.2021.130651.
15. L. Yang, R.M. Liu, L.F. Jiao. Electronic redistribution: construction and modulation of interface engineering on CoP for enhancing overall water splitting. *Adv. Funct. Mater.* **2020**, *30*, 1909618.
16. Liang, H.; Gandi, A.N.; Anjum, D.H.; Wang, X.; Schwingenschlögl, U.; Alshareef, H.N. Plasma-Assisted Synthesis of NiCoP for Efficient Overall Water Splitting. *Nano Lett.* **2016**, *16*, 7718–7725, https://doi.org/10.1021/acs.nanolett.6b03803.
17. Liu, Z.; Yu, X.; Xue, H.; Feng, L. A nitrogen-doped CoP nanoarray over 3D porous Co foam as an efficient bifunctional electrocatalyst for overall water splitting. *J. Mater. Chem. A* **2019**, *7*, 13242–13248, https://doi.org/10.1039/c9ta03201k.
18. Liu, Y.; Zhu, Y.; Shen, J.; Huang, J.; Yang, X.; Li, C. CoP nanoparticles anchored on N,P-dual-doped graphene-like carbon as a catalyst for water splitting in non-acidic media. *Nanoscale* **2018**, *10*, 2603–2612, https://doi.org/10.1039/c7nr07274k.
19. Hua, Y.; Xu, Q.; Hu, Y.; Jiang, H.; Li, C. Interface-strengthened CoP nanosheet array with Co_2P nanoparticles as efficient electrocatalysts for overall water splitting. *J. Energy Chem.* **2018**, *37*, 1–6, https://doi.org/10.1016/j.jechem.2018.11.010.
